# The limits of clinical findings in similar phenotypes, from Carpenter to ATRX syndrome using a whole exome sequencing approach: a case review

**DOI:** 10.1186/s40246-021-00348-x

**Published:** 2021-08-04

**Authors:** Samantha S. Sáenz, Benjamin Arias, Kazuyoshi Hosomichi, Vanessa I. Romero

**Affiliations:** 1 School of Medicine, Universidad San Francisco de Quito, Quito, Ecuador; 2 Onelabt S.A., Ballenita, Ecuador; 3 Department of Bioinformatics and Genomics, Kanazawa University, Kanazawa, Japan

**Keywords:** Rare diseases, ATRX syndrome, Whole exome sequencing

## Abstract

**Background:**

The diagnostic process for uncommon disorders with similar manifestations is complicated and requires newer technology, like gene sequencing for a correct diagnosis.

**Main body:**

We described two brothers clinically diagnosed with Carpenter syndrome, which is a condition characterized by the premature fusion of certain skull bones (craniosynostosis), abnormalities of the fingers and toes, and other developmental problems, for which they underwent craniotomies. However, whole exome sequencing analysis concluded a novel pathological variation in the ATRX chromatin remodeler gene and protein remodeling demonstrated structural variations that decreased the function, giving a completely different diagnosis to these patients.

**Conclusion:**

Our study focuses on the importance of using newer technologies, such as whole exome sequencing analysis, in patients with ambiguous phenotypes.

**Supplementary Information:**

The online version contains supplementary material available at 10.1186/s40246-021-00348-x.

## Background

The diagnostic process for uncommon disorders consists of various steps including the appearance of an unusual characteristic on the individual, the gathering of information, including the clinical history, physical examination, diagnostic testing, and the referral [1]. However, in rare diseases, symptoms and clinical findings are shared between syndromes, which makes the clinical diagnosis uncertain and the necessity for more advanced testing, including gene sequencing. For example, intellectual disability (ID) is an unspecific feature shared in many disorders and is impractical for a specific diagnosis [2]. The diagnostic journey for patients with rare conditions can take a minimum of 1 year to more than 10 years [3]. We present a case of two brothers, from non-consanguineous parents, that physically resemble Carpenter syndrome [MIM:201000], which is a condition characterized by the premature fusion of certain skull bones (craniosynostosis), abnormalities of the fingers and toes, and other developmental problems [4]. Carpenter syndrome can be caused by mutations in the RAB23 or MEGF8 gene and is inherited in an autosomal recessive manner). These children were misdiagnosed for over 9 years and whole exome sequencing (WES) results provide the correct diagnosis.

## Main text

### Patient A, a 10-year-old Hispanic boy

**Fig. 1:**
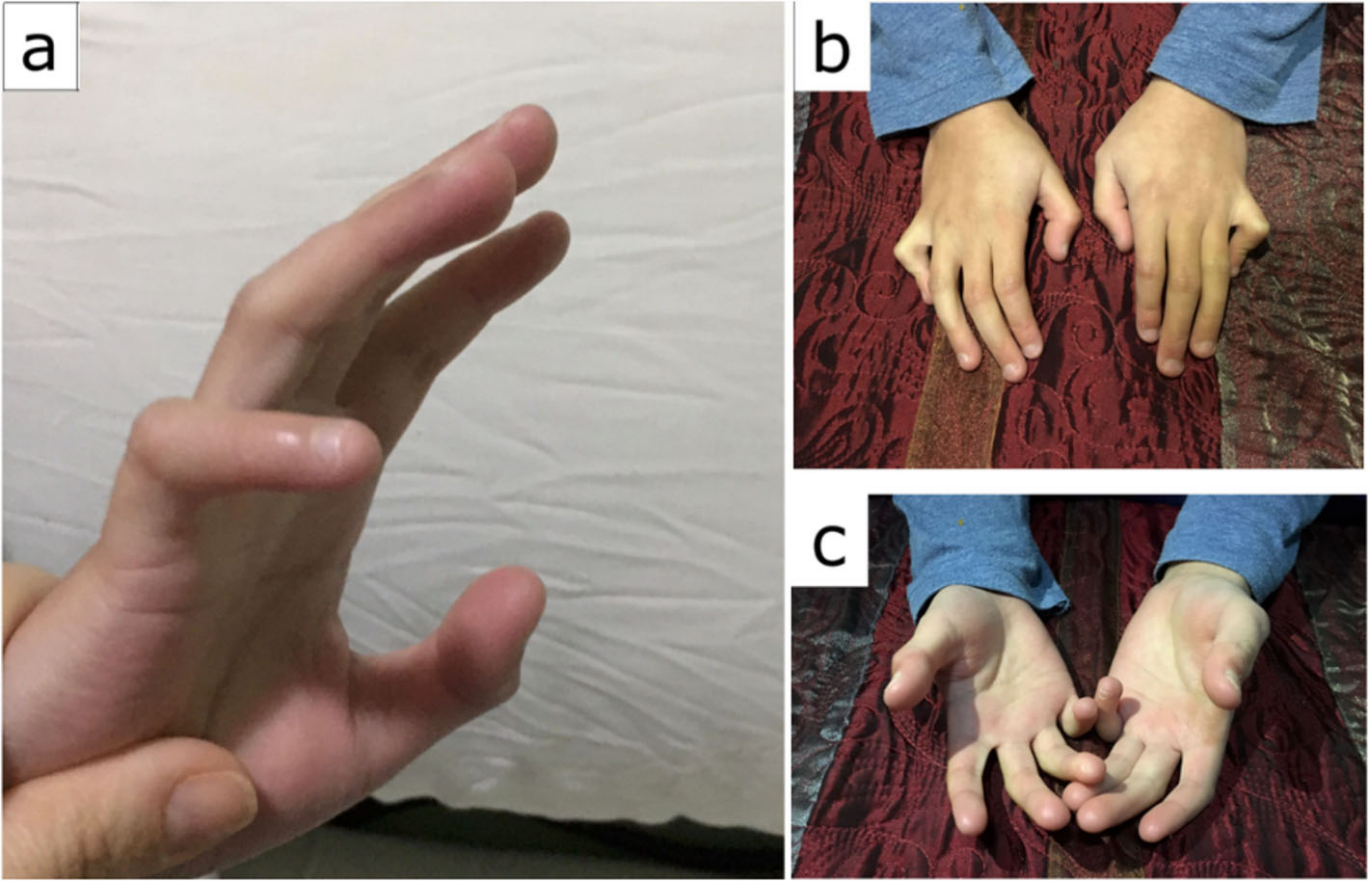
Patient 1 at 11 years old. **a**–**c** Hands abnormalities (clinodatlily, camptodactily, and brachydactlyly)

At 7 months, he was diagnosed with craniosynostosis (sagittal and coronal sutures fused) and underwent surgical correction. Unusual findings at that time included premature teething at 3 months, left inguinal hernia and hip dysplasia, and facial dysmorphia including left-eye strabismus, right-eye ptosis, hypertelorism, wide nasal bridge, and long philtrum. The boy exhibited global developmental delay and verbal aphasia, hand and feet abnormalities (clinodactily, camptodactyly, syndactyly and brachydactyly, and valgus feet), dolichol colon diagnosed by barium enema, and mild valve insufficiency (Fig. 1)

### Patient B, brother of patient A, is a 9-year-old Hispanic boy

**Fig. 2:**
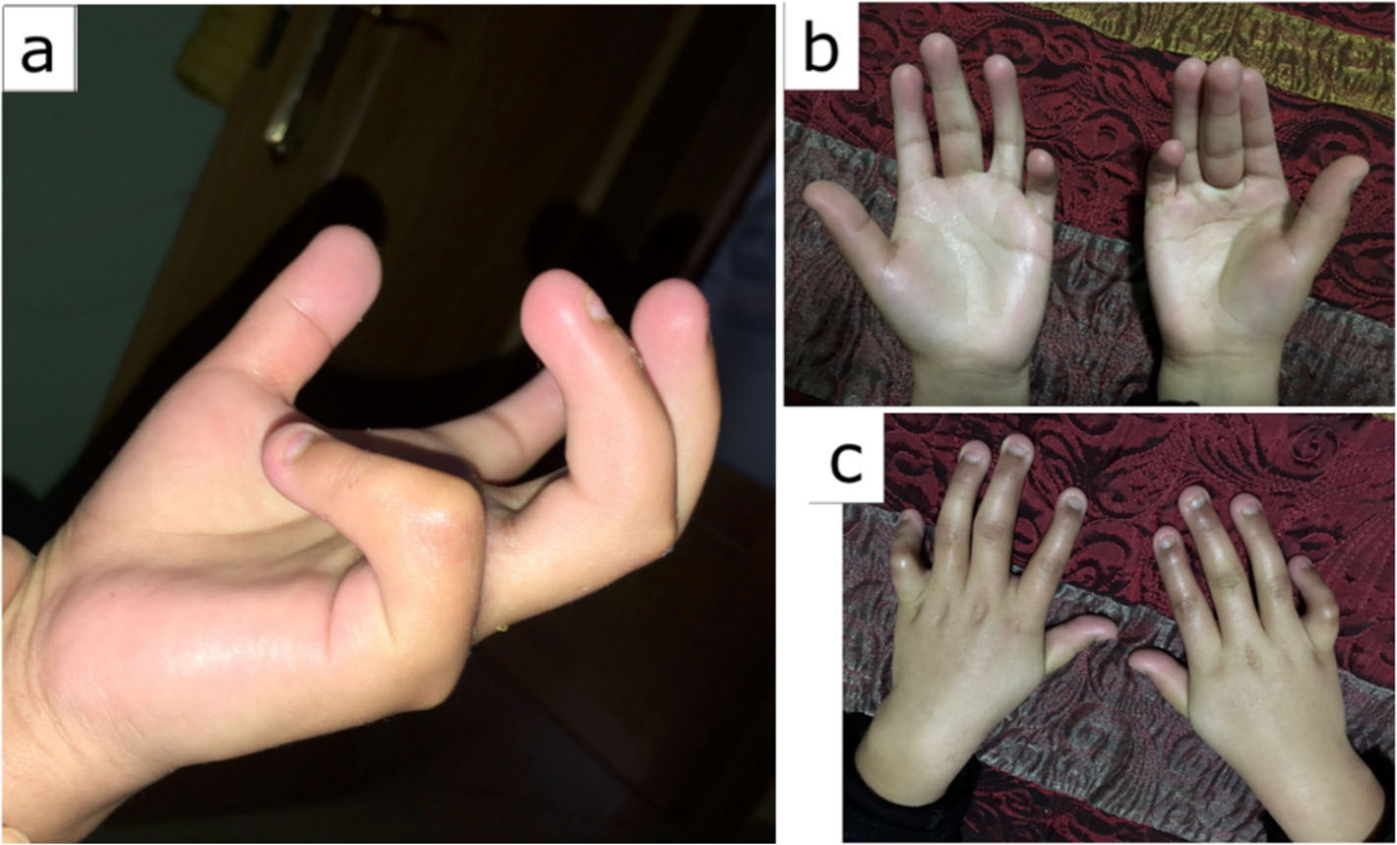
Patient 2 at 10 years old. **a**–**d** Hands abnormalities (clinodatlily, camptodactily, and brachydactlyly)

He was diagnosed with ongoing fusion of the sagittal and coronal sutures at 10 months and underwent surgical correction. Similar to his brother, patient B had dental abnormalities (teething at 5 months), left hip dysplasia, facial, hand, and feet dysmorphias, and global developmental delay. Distinctly, he had malar hypoplasia, dorsal kyphosis, and spina bifida occulta (Fig. 2).

**Fig. 3:**
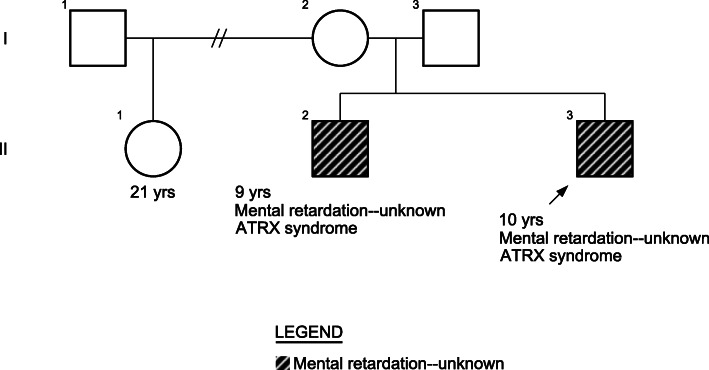
Family Pedigree. It is shown the two brothers and the half-sister with similar physical characteristics but no intellectual disability

Both patients had normal laboratory studies, including blood smear, audiometry, brain CT and karyotype. Chromosome microarray analysis from patient A confirmed no copy number changes or copy neutral regions; focused analysis of the gene *RAB23* [MIM:606144] did not reveal any loss or gain of genetic material. The brothers have a half-sister from the mother’s side with similar physical characteristics, but no intellectual disability. The half-sister was not tested (Fig. 3).

Blood sample from the mother and children were analyzed using the Illumina Miseq sequencing platform. WES analysis revealed a non-synonymous variation found on the mother and the brothers (ATRX chromatin remodeler [MIM:300032]) that affect two isoforms, the first one in methionine to valine in exon 29 (NM_138270:c. 6397A>G p.Met2133Val) and the second one is a methionine to valine in exon 30 (NM_000489:c.6511A>G p.Met2171Val) in chromosome *Xq21.1*. This variant was not reported in ATRX-syndrome (Alpha-thalassemia X-linked intellectual disability syndrome [MIM: 301040]), nor was it present in databases of control individuals (1000 Genomes, ExAC (Exome Aggregation Consortium) and gnomAD (Genome Aggregation Database). To confirm the damage that the variation has on the protein, we decided to perform an open protein structure of the ATRX chromatin remodeler protein. We performed protein modeling of the ATPase-dependent C-terminal Helicase domain of the protein (I-TASSER platform) focusing on the whole secondary structure and the lateral chains [5].

**Fig. 4:**
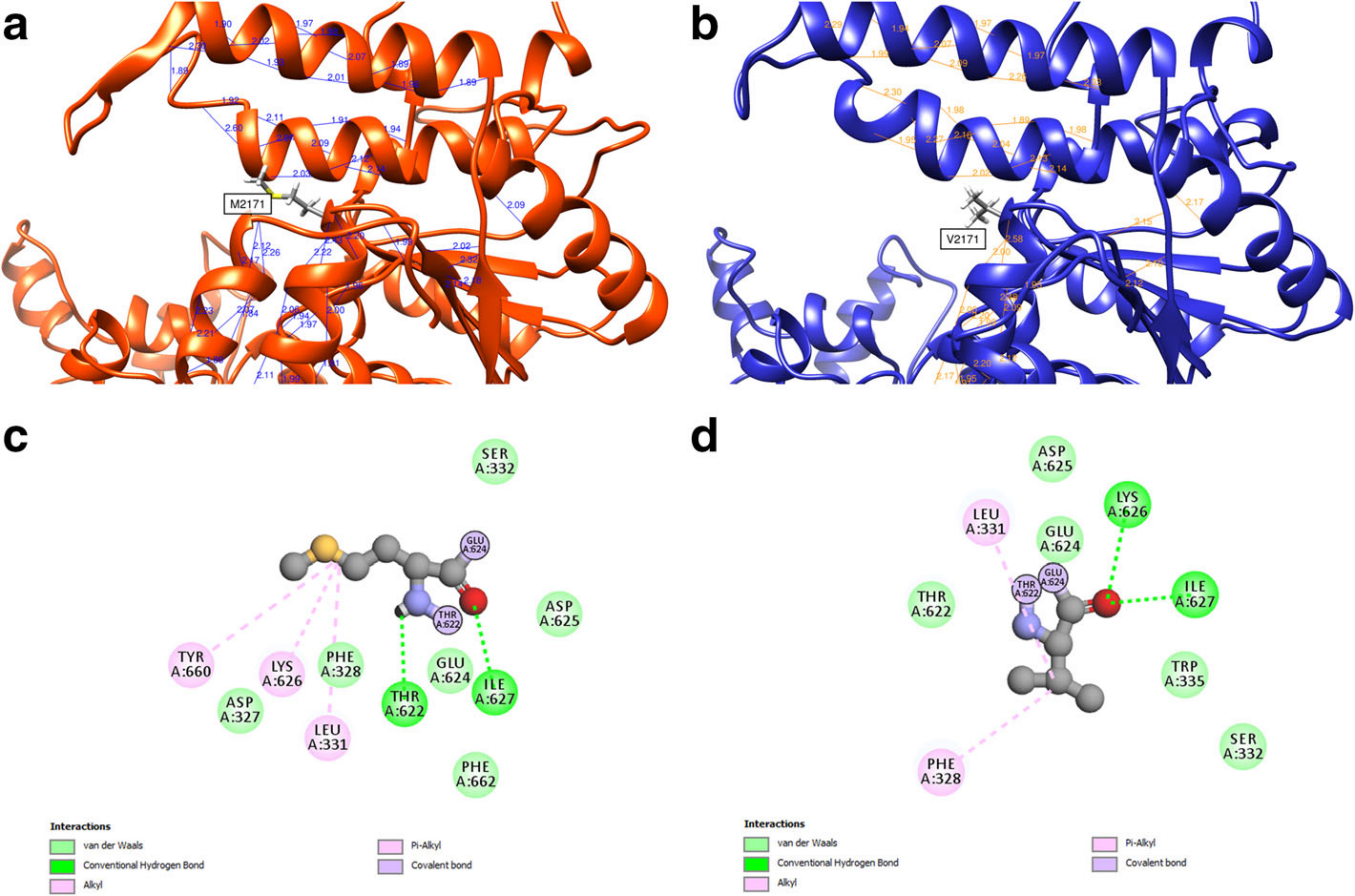
Affected amino acid neighborhood analysis. **a**, **b** Structural neighborhood *Wt* and mutant M2171V respectively. Notice how intrahelical and structural hydrogen bonds change when compare. **c**, **d** One on one bond neighborhood of *Wt* and mutant M2171V respectively. See how differential bond conformation is made with adjacent amino acids

We performed protein modeling of the ATPase-dependent C-terminal Helicase domain (I-TASSER platform) focusing on the whole secondary structure and the lateral chains [5]. Protein modeling allows us to interpret and search structural aberrations that explain the genetic loss of function. Mutation is located on the α-helix which corresponds to the bridge domain between the two catalytical lobes of the SWI/SNF2 chromatin remodeler family [6, 7]. This structure is highly conserved in this family and is well known to act as a negative regulatory domain [8–10]. At the biochemical level, a substitution from methionine to valine supposes a minimal alteration, as the two amino acids are non-polar aliphatic [6]; however, we found that this variation decreases the protein function. Valine’s isopropyl group is a poor binding group and the intra residue covalent bonds activity from the mutant protein differs in Py-Alkyl, Vander Walls and conventional Hydrogen bonds (Fig. 4) [11, 12]. In addition, we identified that at the atomic level (Chimera and Discovery Studio Softwares), the lateral chains are 54.34% similar to the normal protein [13, 14] (Fig. 3, Figure S1, Figure S2, Figure S3). These findings allow us to interpret that the M2171V mutant protein presents different angles inside and between secondary structures of the α-helix which correspond to the bridge domain between the two catalytical lobes of the SWI/SNF2 chromatin remodeler family [6, 7]. This structure is highly conserved in this family and is well known to act as a negative regulatory domain [8–10]. It is important to mention that functional analysis is required to definitively demonstrate the pathogenicity of the variant.

## Discussion

The patients were evaluated after birth due to, what was described in the medical records, as an early fusion of cranial sutures, which can occur either as an isolated event, or syndromic in conjunction with other anomalies (e.g., Carpenter, Apert, Crouzon, Pfeiffer and Saethre-Chotzen syndromes) [15]. Initially, the physicians diagnosed them with Carpenter Syndrome, as they depicted similar clinical characteristics, such as “craniosynostosis,” peculiar facies, hands and feet abnormalities, mental retardation, congenital heart defects and genu valgus. However, they do not have hypogonadism, obesity, cerebral malformations, hydronephrosis, precocious puberty, or hearing loss. Carpenter syndrome is associated with variations in *RAB23 gene*; however, the sibling’s genes did not exhibit this variation. The patients’ diagnosis remained uncertain for 9 years until WES analysis confirmed ATRX syndrome, a condition linked to intellectual disability, dysmorphic facial features and skeletal abnormalities, microcephaly, neonatal hypotonia, genital abnormalities, gonadal dysgenesis, gut dysmotility, and short stature [16]. Genes associated with the other syndromes (e.g., Carpenter [RAB23 gene, MEGF8 gene], Apert [FGFR2 gene], Crouzon [FGFR2 gene], Pfeiffer [FGFR2 gene, FGFR1 gene], and Saethre-Chotzen syndromes [TWIST1 gene]) were analyzed by WES analysis and none of these mutations were found on the patients. It is important to acknowledge that WES analysis other analyses such as chromosome microarray have limitations and some genomic alterations may not be detected, all these exams in conjunction give a definite diagnosis.

The ATRX chromatin remodeler gene is a member of the Switch 2, sucrose nonfermenting 2 (SWI2/SNF2), located on the long arm of the human X chromosome [17] at pericentric heterochromatin domains, and regulates regulating DNA methylation of many target genes in diverse cell types. One of them being a critical transcriptional regulator of globin gene expression, resulting in alpha-thalassemia [17]. Constitutional null mutation is lethal [17, 18]. More than 120 mutations in ATRX chromatin remodeler have been reported and it has diverse clinical manifestations [19, 20]. The ATRX chromatin remodeler is located in the long arm of the X chromosome and is subject to skewed X chromosome inactivation in heterozygote females, which might be the case in the non-tested half-sister [1].

In the case we described, physicians diagnosed the siblings with craniosynostosis and performed the correcting surgery. Ten years after their craniotomy, the patients were analyzed by a geneticist who suggested WES analysis which changed the diagnosis. Table 1 shows how the clinical characteristics present in both patients are shared in different syndromes and emphasizes the importance of using genetic sequencing to differentiate them. Primary care physicians order laboratory tests in about one third of patients, feeling uncertain of interpreting the results in 8% of them [21]. The two most important challenges that physicians face when ordering laboratory tests are: the cost factors; and ordering mechanisms, which refers to different test names, or tests not available except in a panel [21]. Some tactics used to overcome these challenges include curbside consultation, E-reference, and referral to a specialist [21]. Genetic disorders are difficult to confirm using clinical or laboratory criteria, as many are genetically heterogeneous or have variable phenotypes [22]. In such cases, referral to specialists for WES shows the most clinical utility for diagnosis [22]. In Latin American countries there is limited availability to genetic specialists and even more to genetic testing. Many rare diseases require a prompt diagnosis to begin an appropriate treatment, give genetic counseling for the family, and reduce the financial and psychological burden associated with frequent inconclusive medical consultations, as many patients spend many years going from one doctor to another with an uncertain diagnose.

**Table 1:** Characteristics present in our patients that are shown in different craniosynostosis syndromes

Syndromes							
Clinical characteristics	Our patients	Carpenter	Apert	Crouzon	Pfeiffer	Saethre-Chrotzen	ATRX
Premature teething	+	+	+	+			+
Inguinal hernia	+						
Hip dysplasia	+						
Strabismus	+		+	+			
Ptosis	+		+			+	
Hypertelorism	+		+	+	+	+	+
Wide nasal bridge	+						
Long philtrum	+						
Clinodactily	+						
Camptodactyly	+						
Syndactyly	+	+	+		+	+	
Brachydactyly	+	+			+		
Valgus feet	+	+					
Developmental delay	+	+	+			+	+
Verbal aphasia	+						+
Dolichol colon	+						+
Malar hypoplasia	+						
Dorsal kyphosis	+	+					
Spina bifida occulta	+						
Mitral valve insufficiency	+	+				+	
Total (out of 20)	20	7	6	3	3	5	5

## Conclusion

In conclusion, the diagnostic process for rare disorders with similar manifestations should not be limited to clinical characteristics; genomic sequence and protein remodeling should be offered to provide a definite diagnosis and start treatment. We identified a novel non-synonymous variation predicted to produce a mutant protein with different lateral chains and secondary structure resulting in a decreased chromatin remodeler activity. Our study provides further support to the phenotypic variability that exists due to genetic defects.

## Supplementary Information

**Additional file 1: Supplementary Figure **Sequence alignment of Wt and M2171V mutant. Note the total identity of both sequences with the exception of the 2172 position. Moreover, both amino acids present the same biochemical properties as non-polar aliphatic amino acids. **Supplementary Figure S2.** Structural conformation and matching of ATRX chromatin remodeler . a) Wt isoform with secondary structure coloring. In light green α -helixes, in purple β-sheet and coils in with. b) Structural alignment of both models. In orange red Wt and in medium blue M2171V mutant. Note strong differences on some α -helixes. In both arrows represent the point where amino acid change occurs. **Supplementary Figure S3.** Disposition of ATP dependent helicase domain of the ATRX chromatin remodeler structure on nucleosome. At the right, both the mutant M2171V and Wt isoforms of the ATRX chromatin remodeler . Note the position of the punctual amino acid change on ATRX chromatin remodeler structure highlighted in yellow. At the left nucleosome made of different histonic proteins (multicolor protein complex) with DNA around it (grey).

## Data Availability

All data generated or analyzed during this study are available from the corresponding author on reasonable request.

## Abbreviations

ATRX: Alpha-thalassemia X-linked intellectual disability
CT: Computed tomography
DNA: Deoxyribonucleic acid
ID: Intellectual disability
WES: Whole exome sequencing
