# Supplementary material for: The limits of clinical findings in similar phenotypes, from Carpenter to ATRX syndrome using a whole exome sequencing approach: a case review
Source: Hum Genomics. 2021 Aug 4;15:49. doi: 10.1186/s40246-021-00348-x (PMC8336023; doi:10.1186/s40246-021-00348-x)
Supplement: Supplementary file 1 — Additional file 1: Supplementary Figure Sequence alignment of Wt and M2171V mutant. Note the total identity of both sequences with the exception of the 2172 position. Moreover, both amino acids present the same biochemical properties as non-polar aliphatic amino acids. Supplementary Figure S2. Structural conformation and matching of ATRX chromatin remodeler . a) Wt isoform with secondary structure coloring. In light green α -helixes, in purple β-sheet and coils in with. b) Structural alignment of both models. In orange red Wt and in medium blue M2171V mutant. Note strong differences on some α -helixes. In both arrows represent the point where amino acid change occurs. Supplementary Figure S3. Disposition of ATP dependent helicase domain of the ATRX chromatin remodeler structure on nucleosome. At the right, both the mutant M2171V and Wt isoforms of the ATRX chromatin remodeler . Note the position of the punctual amino acid change on ATRX chromatin remodeler structure highlighted in yellow. At the left nucleosome made of different histonic proteins (multicolor protein complex) with DNA around it (grey). [file 40246_2021_348_MOESM1_ESM.pdf]

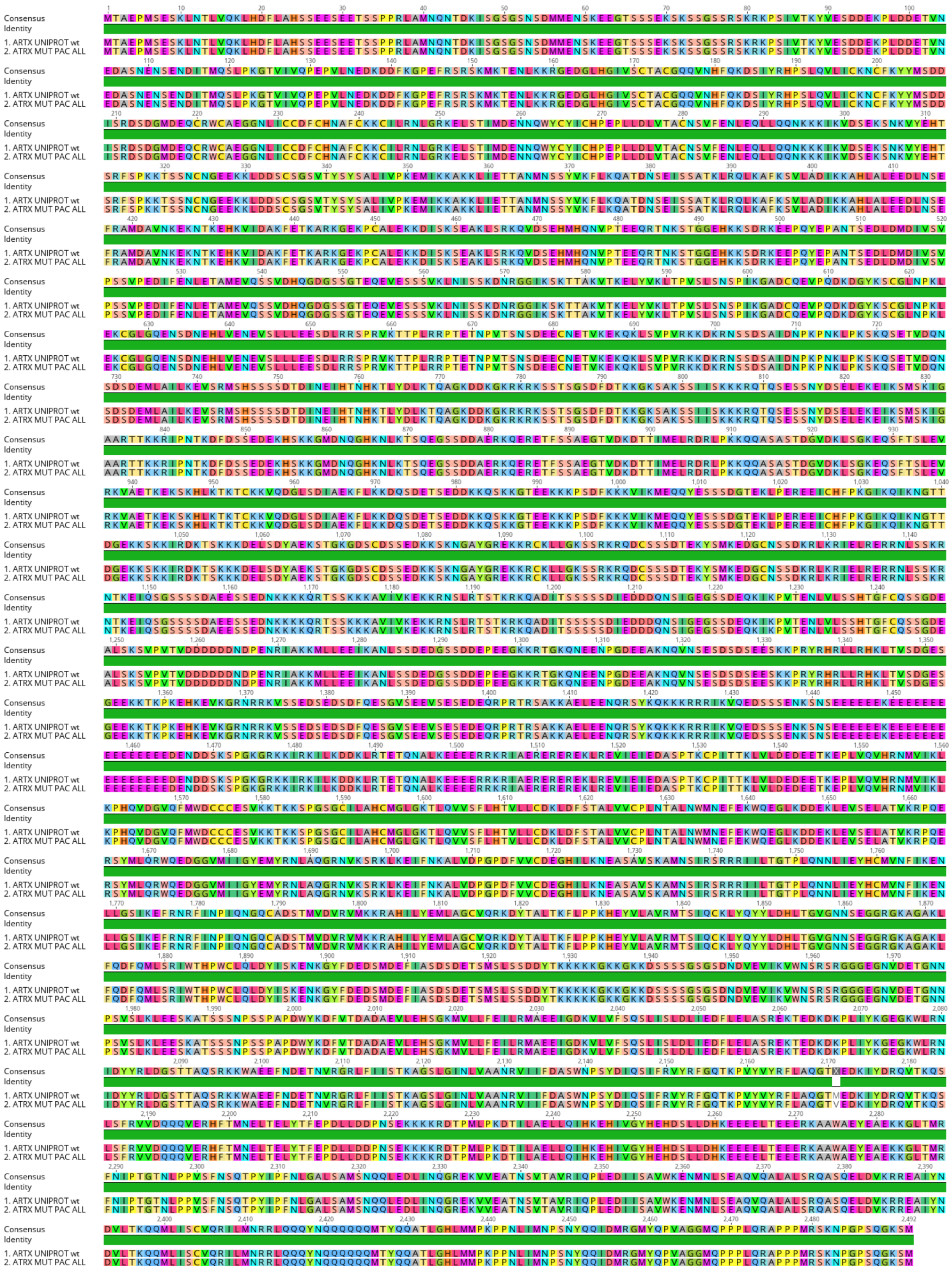

Figure S1. Sequence alignment of *Wt* and M2171V mutant. Note the total identity of both sequences with the exception of the 2172 position. Moreover, both amino acids present the same biochemical properties as non-polar aliphatic amino acids.

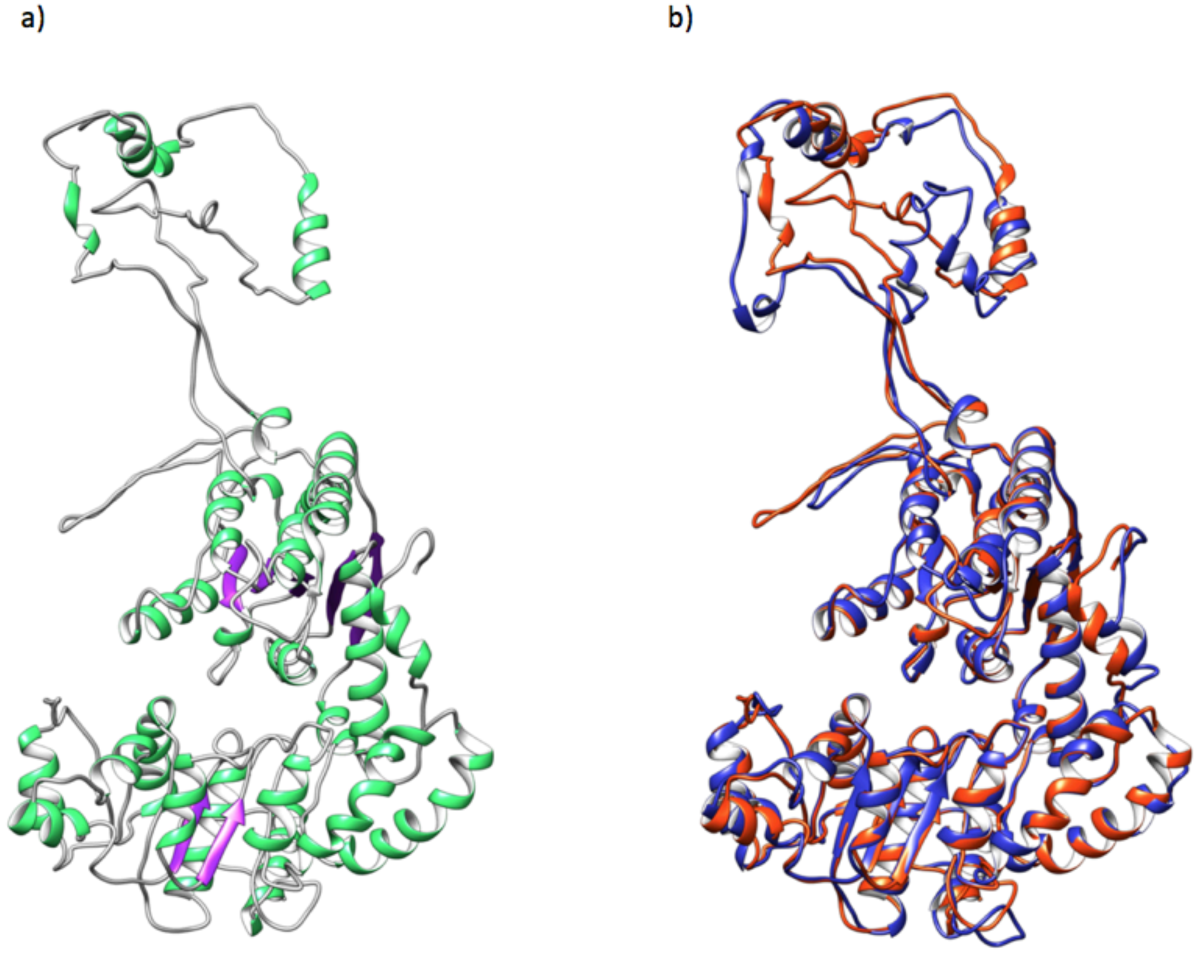

Figure S2. Structural conformation and matching of ATRX. a) *Wt* isoform with secondary structure coloring. In light green α -helixes, in purple β-sheet and coils in with. b) Structural alignment of both models. In orange red *Wt* and in medium blue M2171V mutant. Note strong differences on some α -helixes. In both arrows represent the point where amino acid change occurs.

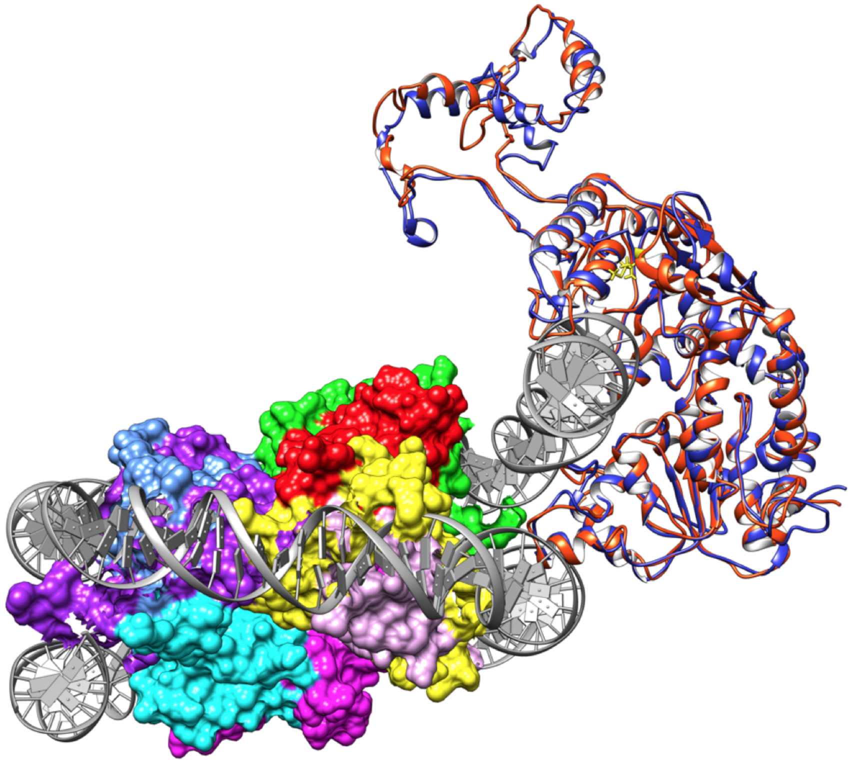

Figure S3. Disposition of ATP dependent helicase domain of the ATRX structure on nucleosome. At the right, both the mutant M2171V and *Wt* isoforms of the ATRX. Note the position of the punctual amino acid change on ATRX structure highlighted in yellow. At the left nucleosome made of different histonic proteins (multicolor protein complex) with DNA around it (grey).
